# We Surrender Space—Alabama State Medical Association

**Published:** 1874-06

**Authors:** 


We surrender space in the present number of the Journal for the proceedings of the late gathering of ex-Confederate Medical Officers in this city.

The meeting, although not so numerously attended as we could have wished, was pleasantly harmonious, and resulted in an organization which we trust will survive in years to come for very many similar reunions. It was not to be expected that the attendance would be large at this meeting, when we consider the very imperfect manner in which it was brought to the attention of those interested, together with the extreme hardness of the times and the exceeding impecuniosity of those who served the Confederacy in its medical department.

The interest manifested by those who did attend, together with the efforts which are to be made to arouse the ex-Confederates during the coming year, will doubtless culminate in a very large and enthusiastic meeting at Richmond.

---

In the May number of the Journal the late meeting of the Alabama State Medical Association is stated as the *sixth* annual session. This is an error, as we learn from the Secretary that the Association was organized in Mobile in 1847.

